# Impact of the Introduction of Accreditation Standards on the Satisfaction of Patients in Cardiology Departments

**DOI:** 10.3390/healthcare9081026

**Published:** 2021-08-11

**Authors:** Rafal Mlynarski, Rafal Kaczkowski, Agnieszka Mlynarska

**Affiliations:** 1 Department of Electrocardiology and Heart Failure, School of Health Sciences, Medical University of Silesia, 40-635 Katowice, Poland; rmlynarski@sum.edu.pl; 2 Upper Silesian Heart Centre, Department of Quality in Health Services, 40-635 Katowice, Poland; akredytacja@gcm.pl; 3 Department of Gerontology and Geriatric Nursing, School of Health Sciences, Medical University of Silesia, 40-635 Katowice, Poland

**Keywords:** hospital accreditation, patient satisfaction, quality of care

## Abstract

Background: It can be presumed that introducing accreditation standards and obtaining national accreditation by a hospital should translate into increased patient satisfaction. The aim was to analyze the impact of introducing accreditation standards on patient satisfaction in cardiology departments. Methods: 1080 patients, who were hospitalized in four cardiological wards (W1–W4) between 2009–2015, were asked to complete a 22-item questionnaire that assessed the level of their satisfaction with their medical care. 58 questionnaires were excluded because of incomplete data. The hospital was accredited in 2013. Results: In 3 of the 4 wards, a statistically higher total score (the patient was more satisfied) in the period after the accreditation (2013–2015) compared to the period before the accreditation (2009–2012) was as follows: W1 (80.37 ± 6.54 vs. 83.85 ± 5.9; *p* = 0.0004), W2 (79.95 ± 7.62 vs. 81.46 ± 8.2: *p* = 0.0376), W4: (78.84 ± 7.94 vs. 84.91 ± 5.57; *p* = 0.0376); in one ward, there was no statistical difference: W3 (80.11 ± 8.42 vs. 81.07 ± 8.15; *p* = 0.3284). A significant difference was found in the number of points for the total assessment that were collected for all of the analyzed departments throughout the entire period (2009–2015)–W1: *p* = 0.0032; W2: *p* = 0.0176; W3: *p* = 0.0313 and W4: *p* < 0.0001). The medium-term rate of the change of the total score decreased after the accreditation. Conclusion: Preparing a hospital for a national accreditation program brought significant benefits for patients in a long-term observation.

## 1. Introduction

The idea of accrediting medical facilities originated in the United States in the 1920s as a reaction of American surgeons to the poor quality of medical services [1]. In Europe, the first accreditation was introduced by the United Kingdom in 1990 [2,3,4]. Poland was the fifth European country to introduce a hospital accreditation system. The organization that was established for this purpose in Poland was the Accreditation Center at the Cracow Center for Quality Monitoring in Health Care (originally the CMJwOZ). Currently, the Center conducts the accreditation process for health care organizations under the auspices of the Minister of Health of Poland [5]. Accreditation is granted for a period of three years–it is focused on the quality and safety of patient care. An accredited hospital provides a message to the community about the good functioning of a hospital and that it meets the highest social expectations. During the accreditation process, almost all aspects related to a patient’s stay in a hospital are assessed [6]. Implementing the accreditation standards should improve the quality of medical services, and thus, patient safety, which should translate into an increase in satisfaction for the main beneficiaries of healthcare–the patients.

Patient satisfaction with medical care is one of the most important components of the operation of the national healthcare system, and thus, justifies the expenditure of public money. Cardiovascular diseases are a leading cause of mortality internationally. When combined, ischemic heart disease and all forms of stroke were the attributed causes of death for an estimated 13 million people globally in 2010 [7]. Therefore, patient expectations are particularly high for the departments that deal with cardiovascular diseases.

It can be presumed that obtaining national accreditation by a hospital should translate into increased patient satisfaction. There are differences in the research on the impact of accreditation on patient satisfaction, e.g., Sack et al. found that while hospital accreditation may represent a step towards quality management, it does not seem to improve overall patient satisfaction [8]. On the other hand, Devkaran et al. concluded that the improvements that are achieved during the process of accreditation are maintained during the three-year accreditation cycle [9]. The purpose of this study was to assess the impact of introducing the accreditation standards of the Monitoring Centre for Quality in Health Care on patient satisfaction in the cardiology departments of the leading cardiology centers in Poland.

## 2. Materials and Methods

This prospective study included 1080 patients who were hospitalized in the four cardiology departments of the Medical Center between 2009–2015. All of the enrolled patients were asked to complete a questionnaire that assessed the level of their satisfaction with their medical care. The patients who were enrolled in the study were selected by the Ward Nurse from among patients that were scheduled to be discharged from a given ward in the period of the analysis. On the day of discharge, the enrolled patients received the author’s standard, anonymous, permanent survey questionnaire. The entire questionnaire consists of 13 questions with sub-questions, for which the points are summed during the analysis–from 1–2 to 1–5 points depending on the question. A total of 22 issues are assessed in the questionnaire. The issues that are covered by the questionnaire are presented in the Appendix A. A higher number of points means a higher level of patient satisfaction with their care. The accuracy of the questionnaire was confirmed in the studied population and will be the subject of a separate publication. Finally, the completed questionnaires were collected from 1022 patients of the 1080 patients. The questionnaires of 58 patients were excluded from the study because of incomplete data.

Thirty patients per year/per ward were included in the years 2009–2012 and 50 patients per year/per ward in the years 2013–2015, who were hospitalized in the four departments with a cardiological profile of a conservative-invasive subtype. The names of the analyzed departments were randomly coded under the names W1, W2, W3 and W4.

This study was approved by Medical University of Silesia Bioethical Committee.

### 2.1. Accreditation Process

The researched hospital was accredited in the first half of 2013. The set of accreditation standards of the Monitoring Centre for Quality in Health Care imposes on hospital departments the need to set quality indicators and conduct the following analyzes:

- Analysis of the early effects of the performed procedures
- Analysis of the long-term effects of the performed procedures (follow-up)
- Analysis of the reasons for the extended stay of patients
- Analysis of the causes of patient deaths, including the causes of perioperative deaths
- Bedsore analysis
- Analysis of adverse events related to the hospitalization
- Analysis of unplanned, repeated hospitalizations
- Analysis of the content, completeness and authorization of medical records, including the frequency of polypragmasy cases and the validity of the drugs used
- Monitoring the outcomes and complications of enteral nutritional therapy and intravenous nutritional therapy
- Hospital infection monitoring and infection data validation

The implementation of the standards began in accordance with the Implementation Schedule developed in the last quarter of 2010. For this purpose, the following entities were appointed to support the implementation of standards and the development of the accreditation program:

- Quality control commission
- Commission for the analysis of treatment effectiveness
- Pain treatment committee
- Committee for the analysis of anesthesia and resuscitation
- Committee for solving of ethical issues
- Committee for the development of health-related procedures
- Committee for the development of standards for the prevention of thromboembolism
- Team for developing procedures to be followed in the event of a multiple, mass incident and catastrophe
- Strategic plan development team

### 2.2. Statistical Analysis

The data were entered into a relational database that was designed to specifically meet the needs of this study using the MySQL database platform and the data was processed using the SQL 92 query language. Before counting the total number of points that were collected for the patients’ responses, questions with incomplete answers or 0 = no need or 0 = not applicable were excluded from the database. The questions that were analyzed in the questionnaire were used to estimate the number of points for the total assessment. Thus, the minimum total score was 25 points, while the maximum total score was 90 points. The reliability of this questionnaire was demonstrated in the studied population in an earlier preliminary study.

The statistical analysis was performed using the MS Excel (Microsoft Corporation Redmond, Washington, U.S.) software package. To analyze the impact of accreditation on a specific question, the average value of the points in 2009–2012 (the period before the accreditation) and 2013–2015 (the period after the accreditation) were calculated. The Mann-Whitney U test was used to compare the number of points for the total assessment during the period after (2013–2015) compared to the period before (2009–2010). The Kruskal-Wallis ANOVA test was used to statistically assess the differences in the number of points in the total assessment in consecutive years. The significant results of the Kruskal-Wallis test required multiple comparisons (post-hoc tests), which made it possible to answer the question of which of the analyzed groups differed from one another. It was assumed that the results that were collected were significant at a *p* value < 0.05. The medium-term rate of change was also analyzed for each of the wards. To do this, in the first stage, the so-called chain indexes divided a value from a given year by the value for the previous year, while in the next stage, the mean iG index, the geometric mean of six chain indexes and the average periodic rate of change T, was calculated. The medium-term rate of change could be positive or negative. If the value T > 0, it meant that the average increase of the studied issue from period to period, i.e., if the value of T for a given variable was +7.77% (a value greater than zero), it meant that on average from year to year (in 2009, 2010, 2011, 2012, 2013, 2014 and 2015), the average value of this variable increased by about 7.8%. If T < 0, then, there was a decrease in the average rate of that phenomenon.

## 3. Results

### 3.1. Descriptive Analysis

The average value from the questionnaire was 81.6 points, which was significantly higher than the hypothetical calculated value of 57.5 points (*p* < 0.0001). This indicated a high-positive assessment of the factors that were analyzed in the questionnaire in the surveyed hospital departments throughout the entire study period.

The distributions of the number of points for the total assessment for all of the analyzed wards are presented in Table 1. Based on the results of the Kruskal-Wallis ANOVA test, there was a statistically difference in the number of points in the total score for Ward 1 throughout the entire study period (2009–2015) (*p* = 0.0032). Statistical differences were also found in Ward 2 (*p* = 0.0176), in Ward 3 and in Ward 4 (*p* < 0.0001).

**Figure 1:**
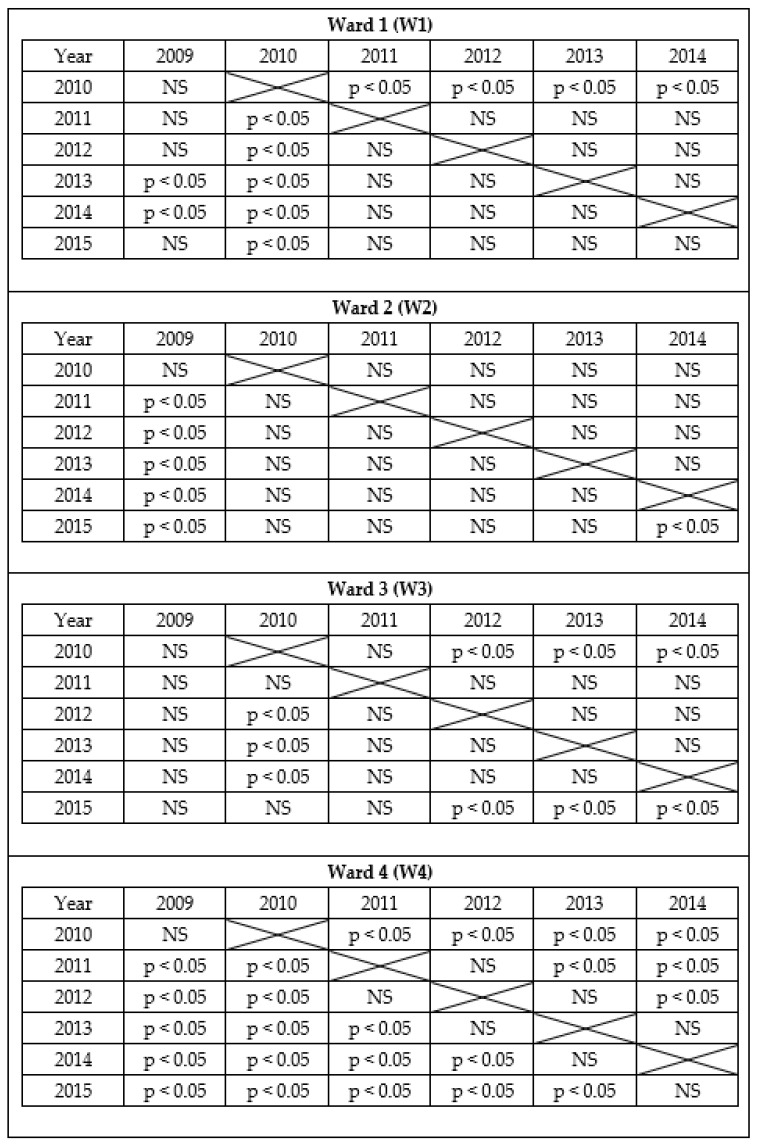
Total assessment between the subsequent years based on average ranks using the post-hoc test.

Additionally, the total assessment between the subsequent years based on average ranks using the post-hoc test are presented in the Figure 1. In 2009, the number of points for the total assessment for Ward 1 was statistically lower than in 2013 and 2014 and that in 2010, it was statistically lower than in 2011, 2012, 2013, 2014 and 2015. For Ward 2 in 2009, the number of points for the total assessment was statistically significantly lower than in 2011, 2012, 2013, 2014 and 2015. On the other hand, in 2010 and in 2015, the number of points for the total assessment for Ward 3 was statistically significantly lower than in 2012, 2013 and 2014. Lastly, in 2009 and 2010, the number of points for the total assessment for Ward 4 was statistically significantly lower than in 2011–2015; in 2011, it was lower than in 2013–2015; in 2012, it was lower than in 2014 and 2015 and in 2013, it was lower than in 2015.

### 3.2. Before vs. after Accreditation Comparison

Patient satisfaction in three departments improved significantly in the post-accreditation period. Only in one ward (W3) the satisfaction of the patients not achieve statistical significance. The selected descriptive statistics and distributions of the number of points in the summary assessment for all of the analyzed wards is presented in Table 2.

### 3.3. Medium-Term Rate of Change

The medium-term rate of change of the total score is presented in Table 3 it can be seen that this rate decreased after accreditation. What is more important is that in two wards (W1 and W3), this rate had a negative value.

## 4. Discussion

The effectiveness of healthcare is the most important element of a medical community. Patient satisfaction with the medical care that is offered to them is equally important [10]. This satisfaction is also the most important factor for the cooperation of a medical doctor, a patient and other medical personnel, especially during the clinical follow-up period [11,12]. Satisfaction is a sense that certain expectations have been met. In the case of a patient’s contact with the national health system, many factors affect patient satisfaction that are not related to the success of the medical procedure that was performed, but also their contact with the personnel, the environment in which the patient is treated, the applicable procedures, etc. An important question is whether it is possible to assess a patient’s satisfaction with their medical care, and if so, what methodology should be used? In this publication, we decided to tackle this task and created a tool to assess the overall satisfaction of hospital care. As a result, the original questionnaire that is presented in this paper was created. There are not many tools available to study patient satisfaction in the world. Another example of this is the research of Moon et al. in which the authors developed a Patient Satisfaction Survey for Comprehensive Medication Management [13]. Their tool is composed of ten items that are associated with three domains using a 4-point scale: medication-related needs, pharmacist-patient engagement and overall satisfaction. It is an interesting tool for analyzing patient satisfaction, but compared to our survey, the tool is specialized in one important field–Comprehensive Medication Management, while the survey we propose was designed to assess the overall satisfaction of hospitalized patients.

After assessing and confirming the reliability of the questionnaire that was developed by our team, we decided to also create a model for using the questionnaire. We have listed numerous factors that can affect patient’s satisfaction, but the question of how these factors influence overall satisfaction is still valid. In our opinion, the national accreditation of hospitals is a very useful tool that can increase patient satisfaction. In Poland, this is performed by the Center for Quality Monitoring in Health Care (originally CMJwOZ), which is located in Cracow and is accepted by the Polish Ministry of Health. There is a widespread belief that obtaining such an accreditation should translate into quality health care and an increase in patient satisfaction. But belief is not scientific evidence and we tried to change this presumption into scientific proof. That is why we selected four conservative surgical departments in the field that is broadly understood as cardiology and examined how patient satisfaction differed before and after the accreditation process. Based on the results, there was a significant improvement in the number of points in the total score for all of the analyzed wards. When we compared the periods before and after accreditation, there was also a statistical improvement in patient satisfaction in 3 of the 4 wards.

Interestingly, even in period before accreditation the process of preparation cause improvement in patients’ satisfaction. In the years 2009–2010, the Hospital operated in a “normal” manner, without any challenges related to the implementation of pro-quality standards. In the last quarter of 2010, the Director of Hospital decided to start implementing the accreditation standards of the Monitoring Centre for Quality in Health Care in order to join the accreditation visit and obtain the status of an Accredited Hospital awarded by the Minister of Health of Poland. From the beginning of 2011, the implementation of the standards began in accordance with the Implementation Schedule developed in the last quarter of 2010. For this purpose, some entities were appointed to support the implementation of standards and the development of the accreditation program. The intensification of the activities of the above-mentioned Commission and Implementation Teams was reflected in the progressive increase in the quality results in 2011–2012. It should be noted that the Hospital was preparing for the accreditation visit, which was initially supposed to take place in 2012, but for reasons beyond our Hospital’s control, the certification audit of the Hospital Accreditation Program took place in 2013. We can conclude that preparing for accreditation and obtaining the Minister’s certificate are very beneficial for increasing patient satisfaction with health care. However, further analysis of the results led to a less favorable conclusion. The analysis of the medium-term rate of change of the total score showed that obtaining the certificate itself decreases the rate of the improvement in patient satisfaction with health care. It seems that this unfavorable phenomenon may be avoided by an increase in the frequency of revaluation visits.

We could only find a few similar researches in the world. The most similar in the study design was the research that was performed by Sack et al. in 2010 in which 3037 consecutive patients who were being discharged from 25 specialized cardiology units in Germany completed a validated patient satisfaction questionnaire [8]. The likelihood of recommending the hospital was used as the main endpoint, which is similar to our research. Of the patients, 1835 were treated in an accredited unit, while 1202 were treated in non-accredited units. The Picker Inpatient Questionnaire, which assesses seven dimensions of patient satisfaction including: physician-patient relations, nurse-patient relations, treatment success, quality of accommodation, quality of catering, admission procedure, discharge procedure, perceived cleanliness, inclusion of relatives/friends, and finally, the atmosphere of the relations were assessed [14]. Here, we found the first difference between our study and the one quoted–firstly, the difference between the Picker Inpatient Questionnaire and ours is that some of areas were only covered by one or the other. The patient satisfaction in this research indicated no significant advantage for the accredited units–the scores of the institutions with accreditations (65.6%) were not significantly different from the institutions without a formal accreditation (65.8%) [8]. The authors cited above concluded that obtaining accreditation is not associated with a higher standard of patients care, however, it can support the hospital management process. The impact of hospital accreditation on quality measures was also assessed by Devkaran et al. for a multispecialty hospital in Abu Dhabi in United Arab Emirates [9]. The differences in the quality analysis were compared in monthly ranges for twenty-seven variables describing the quality processes in hospital. Comparative analysis was carried out for one-year accreditation (2009) and three-year period after accreditation (2010, 2011 and 2012) [9]. This methodology is generally similar to the one presented by us, but we used more significant periods of time. The results of the cited study showed that preparation for the accreditation study brought significant improvements-this is in line with our results. The authors quoted different results in the assessment of the period after accreditation-accreditation had a more negative than positive impact on the results of accreditation. In addition, accreditation had no significant impact on 11 of the 27 variables. Authors underlined that there are other benefits of accreditation during a three-year analysis [9].

The authors concluded that although there was a temporary drop in performance immediately after accreditation, some improvement achieved through accreditation was maintained during the three-year accreditation cycle-this unfavorable phenomenon was also demonstrated by us [9].

The conclusions that can be drawn after an analysis of our research and those cited from similar areas of interest are not uniform. In our study, national accreditation had a really positive effect on patient satisfaction, while in the others that were cited earlier, this statement was ambiguous. In our opinion, the discrepancies have resulted from the use of different methodologies and endpoints as well as the basic state of the national health care in different countries, but we still believe that hospital accreditation has positive influence on different areas of hospital functioning including patient satisfaction.

### Limitations of the Study

The aim of the study was not to make comparisons between departments, but to present them as four examples of units that are managed differently with a certain degree of reliability.

The paper does not contain any precise baseline characteristics of the patients included. This is due to the fact that we wanted to maintain the feeling of as much anonymity and comfort as possible when answering the questions. It seems that the introduction of such surveys and the collection of such data may also influence patient responses.

There are also a number of other confounding factors, such as changes in the ratios of medical staff (nurses/doctors) over time or an increase in their education, among others which was not the subject of analysis.

## 5. Conclusions

The process of preparing for and obtaining the national accreditation certificate itself had a positive effect on the selected parameters of patient satisfaction with the four cardiology departments of the Cardiology Center in Poland. The results indicated a difference in the behavior of staff during the post-accreditation period, which suggests the need for continuous external monitoring and not just a periodic accreditation visit.

## Figures and Tables

**Table 1:** Selected descriptive statistics, the number of points for the total assessment for the analyzed departments according to years of analisis.

	2009	2010	2011	2012	2013	2014	2015
	Before Acreditation				After Acreditation		
Ward 1	80.67 ± 6.31	77.83 ± 6.34	83.35 ± 5.59	81.87 ± 7.05	84.06 ± 5.44	84.09 ± 6.48	83.44 ± 6.07
Ward 2	76.14 ± 7.89	80.67 ± 7.02	80.62 ± 8.29	81.77 ± 6.53	82.54 ± 6.80	82.32 ± 7.69	82.90 ± 7.97
Ward 3	78.68 ± 9.37	75.72 ± 10.21	81.81 ± 6.20	83.21 ± 6.21	82.54 ± 6.80	82.32 ± 7.69	78.34 ± 9.22
Ward 4	75.50 ± 6.75	76.40 ± 6.75	80.93 ± 7.16	81.79 ± 7.51	83.57 ± 6.69	85.16 ± 4.85	85.98 ± 4.82

**Table 2:** Selected descriptive statistics, the number of points for the total assessment before vs. after accreditation for the analyzed departments.

	Before Accreditation	After Accreditation	*p*
Ward 1	80.73 ± 6.54	80.73 ± 83.85	*p* = 0.0004
Ward 2	79.95 ± 7.62	81.46 ± 8.16	*p* = 0.0376
Ward 3	80.11 ± 8.42	81.07 ± 8.15	*p* = 0.3284 NS
Ward 4	78.84 ± 7.49	84.91 ± 5.57	*p* = 0.0376

**Table 3:** Medium-term rate of change of the total score.

	Before Accreditation	After Accreditation	Entire Group
Ward 1	0.49%	−0.37%	0.57%
Ward 2	2.41%	0.22%	1.43%
Ward 3	1.88%	−2.58%	−0.07%
Ward 4	2.70%	1.43%	2.19%

## Data Availability

Data sharing not applicable.

## Appendix A

**Table A1:** Questions (variables) and possible scoring that were used to determine the total score.

Question Number and Short Name of the Question (Variable)	Possible Scoring
2. TIME- hospital admission room	1, 2, 3 or 4
3A. Courtesy of the DOCTOR’S in hospital admission room	2, 3, 4 or 5
3B. Courtesy of the NURSE in hospital admission room	2, 3, 4 or 5
3C. Courtesy of the REGISTRAR (OFFICE PERSONEL) in hospital admission room	2, 3, 4 or 5
4. Doctor’s personal details	Yes or no
5. Doctor’s availability	1, 2 or 3
6A. Assessment of medical care. CAREFULLY LISTENING	1, 2, 3, 4 or 5
6B. Evaluation of medical care PRESERVATION	1, 2, 3, 4 or 5
6C. Assessment of medical care. KINDNESS	1, 2, 3, 4 or 5
6D. Assessment of medical care INTIMACY	1, 2, 3, 4 or 5
7A. Comprehensive medical information about full health information	1, 2, 3, 4 or 5
7B. Comprehensive medical information about methods of treatment	1, 2, 3, 4 or 5
7C. Comprehensive medical information about side effects	1, 2, 3, 4 or 5
7D. Comprehensive medical information about patient’s rights	1, 2, 3, 4 or 5
8. Nursing care evaluation	1, 2, 3, 4 or 5
9. Personel having identifier	Yes or no
10A. Information prior to discharge about continuation of treatment	1, 2 or 3
10B. Information prior to discharge about health prophylaxis	1, 2 or 3
10C. Information prior to discharge about diet	1, 2 or 3
10D. Information prior to discharge rehabilitation	1, 2 or 3
11. Hospital recommendation	1, 2, 3, 4 or 5
12. I like the hospital	Yes or no
13. I was annoyed	Yes or no
